# Marine Structure Derived Calcium Phosphate–Polymer Biocomposites for Local Antibiotic Delivery

**DOI:** 10.3390/md13010666

**Published:** 2015-01-20

**Authors:** Innocent J. Macha, Sophie Cazalbou, Besim Ben-Nissan, Kate L. Harvey, Bruce Milthorpe

**Affiliations:** 1 School of Chemistry and Forensic Science, University of Technology Sydney, Ultimo NSW 2007, Australia; E-Mail: innocent.macha@uts.edu.au; 2 CIRIMAT Carnot Institute, CNRS-INPT-UPS, University of Toulouse, 31030 Toulouse, France; E-Mail: sophie.cazalbou@univ-tlse3.fr; 3 The ithree Institute, Faculty of Science, University of Technology Sydney, Broadway NSW 2007, Australia; E-Mail: Kate.l.harvey@student.uts.edu.au; 4 Faculty of Science, University of Technology Sydney, Broadway NSW 2007, Australia; E-Mail: Bruce.Milthorpe@uts.edu.au

**Keywords:** drug release, thin film composites, coral, hydroxyapatite, microbial adhesion, *S. aureus*

## Abstract

Hydrothermally converted coralline hydroxyapatite (HAp) particles loaded with medically active substances were used to develop polylactic acid (PLA) thin film composites for slow drug delivery systems. The effects of HAp particles within PLA matrix on the gentamicin (GM) release and release kinetics were studied. The gentamicin release kinetics seemed to follow Power law Korsmeyer Peppas model with mainly diffusional process with a number of different drug transport mechanisms. Statistical analysis shows very significant difference on the release of gentamicin between GM containing PLA (PLAGM) and GM containing HAp microspheres within PLA matrix (PLAHApGM) devices, which PLAHApGM displays lower release rates. The use of HAp particles improved drug stabilization and higher drug encapsulation efficiency of the carrier. HAp is also the source of Ca^2+^ for the regeneration and repair of diseased bone tissue. The release profiles, exhibited a steady state release rate with significant antimicrobial activity against *Staphylococcus aureus* (*S. aureus*) (SH1000) even at high concentration of bacteria. The devices also indicated significant ability to control the growth of bacterial even after four weeks of drug release. Clinical release profiles can be easily tuned from drug-HAp physicochemical interactions and degradation kinetics of polymer matrix. The developed systems could be applied to prevent microbial adhesion to medical implant surfaces and to treat infections mainly caused by *S. aureus* in surgery.

## 1. Introduction

Current increases in the ageing population and increased longevity due to medical advances in many developed countries has led to a rise in the number of musculoskeletal disorders (MSDs). The number of medications to prevent and treat these diseases has also expanded in recent times due to scientific advances. The development of new drugs and active substances allows treatment of some of these diseases in very early stages. The key issue that has been explored widely in recent times with regards to these treatments is the ability to direct drugs to specific organs and musculo-skeletal sites. Most importantly, these treatments are designed to be able to effect locally when required. They are required to control the release rate of the drugs, in order to maintain a desired drug concentration levels without reaching to a toxic level or dropping below a minimum effective level. For these reasons, a major effort has been directed on the development of biodegradable materials that are capable of releasing drugs by reproducible and predictable kinetics [1,2].

Drug delivery technology presents an interesting interdisciplinary challenge for pharmaceutical, chemical engineering, biomaterials and medical communities [3]. In general, a biomaterial that will act as a drug carrier must have the ability to incorporate a drug, to retain it in a specific site, and to deliver it progressively with time to the surrounding tissues. Furthermore, it would be advantageous if the material is injectable or alternatively coatable on an implant and most importantly biodegradable.

It has been reported that calcium phosphate bone substitutes derived from mixed hydroxyapatite and β tricalcium phosphate (β-TCP) are the most promising materials for bone drug delivery systems [4]. During the last decade there have been several studies on both commercial and experimental calcium phosphate drug carriers [1]. Major attention has been focused on the delivery of antibiotics, due to their wide areas of applications as prevention against infection during surgical interventions or in general in the treatment of bone infections.

Ceramics and other materials have been proposed in the past, but it is difficult to form an appropriate shape with adequate micro porosity in order to be fitted into any type and size of bone defect. The treatment of bone infection remains difficult because of problems with local penetration of systemically administered antibiotic. Furthermore, bacteria adhere to bone matrix and orthopaedic implants, eluding host defences by developing a biofilm or acquiring a very slow metabolic rate [5]. Effective treatment against infection may be possible by killing the bacteria during the early stages of colonization, followed by the continuous long time steady state delivery of appropriate amounts of antibiotics.

Recently it has been demonstrated that marine shells with specific microspherical design offer desired functions for the delivery of Bisphosphonate (paminodrate) and antibiotic (Gentamicin). This has been possible by virtue of its unique structure and architecture of the foraminifera shells which are extraordinarily difficult to manufacture with the current know-how [6].

Biodegradable polymer films loaded with gentamicin have been developed to serve as “coatings” for fracture fixation devices and prevent implant-associated infections [7]. The use of biodegradable polymer films is advantageous due to its propensity to uptake and release antibiotics, as a consequence of its degradability. Although their drug release rates are high, they could be tailored to form biocomposites with different biodegradability rates by incorporating other materials. Biodegradable polymer-bioceramic composites would be ideal in this endeavour because of the bioactive nature of ceramic materials which promotes tissue growth. Incorporation of bioceramics in these films will improve not only controlled drug release but also bioactivity and tissue regeneration, especially in orthopaedic and maxillofacial applications. Sampath *et al.* [8] demonstrated that PLA microcapsules could release more than 80% of loaded gentamicin sulphate within 3 weeks for the treatment of osteomyelitis. Drug release time and the shape of the release systems would limit their applications especially for prolonged release. Bioerodable, Polyanhydride-Gentamicin beads were used *in vivo* study for the treatment of Osteomyelitis by surgical debridement by Nelson *et al.* [9]. They reported to have eradicated osteomyelitis by 93% after 4 weeks of implantation for 20% gentamicin loaded beads. However most of these past published investigations have shown that antibiotics have been ototoxic and nephrotoxic at high dosages. For most controlled release systems, the loaded dosages are usually high, and therefore the systemic exposure of antibiotic in blood and urine is the major safety concern. Moreover none of these reported systems contain the key minerals like Ca^2+^ to support bone repair and regeneration. In this research, converted coralline hydroxyapatite (HAp) particles loaded with medically active drugs and substances were used to develop polylactic acid (PLA) thin film composites for slow drug delivery systems.

## 2. Results and Discussion

### 2.1. Results

#### 2.1.1. Susceptibility Testing and Drug Encapsulation Efficiency (DEE)

The Minimum Inhibitory Concentration of Gentamicin against *S. aureus* (SH1000) was found to be 1 μg/mL. DEE was determined to be 94.2% and 98.9% for PLAGM and PLAHApGM films respectively.

#### 2.1.2. Drug Loading to HAp

**Figure 1:**
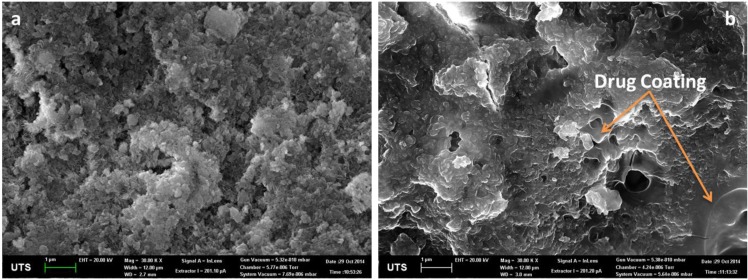
(**a**) Hydrothermal converted coralline HAp; (**b**) Gentamicin coated coralline HAp.

Scanning electron microscopy (SEM) was used to analyze drug loading into HAp particles. Figure 1 shows images of HAp before and after drug loading. It was evident that converted coral particles are coated with drug into micro, meso and nanopores as drug solution could easily penetrate into these pores. On the other hand the surface of HAp before loading reveals thin platelets of HAp crystals while after loading the surface looks relative smooth due to drug coating.

#### 2.1.3. Tensile Strength and Elongation at Break of Composites

**Figure 2:**
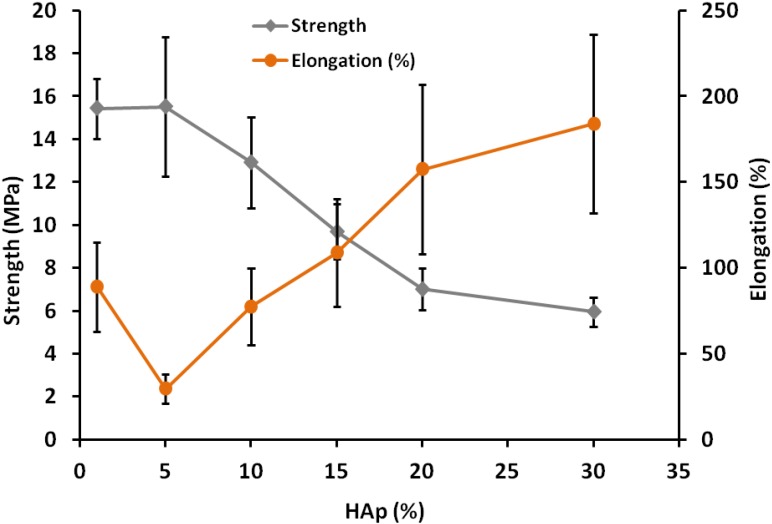
Mechanical properties of PLAHAp composites.

Figure 2 represents mechanical properties of PLAHAp composites at different HAp compositions. Tensile strength decreases as HAp content increases due to poor interfacial bond between PLA and Hap particles. On the other hand elongation increase as HAp contents increases suggesting the increase of composite flexibility due to introduction of voids within the matrix [10].

#### 2.1.4. *In-Vitro* Gentamicin Release

**Figure 3:**
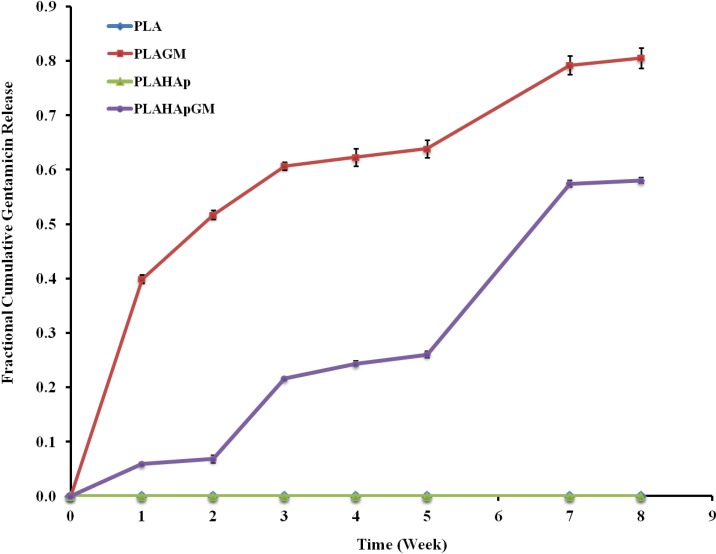
Fractional cumulative release of gentamicin from polylactic acid (PLA) thin film composite in phosphate buffered saline (PBS) solution (pH 7.4, 37 °C and 100 rpm), for eight weeks. Error bars are mean standard deviation (SD) of triplicate experimental data.

The central objective of a delivery system is to release therapeutics at the desired anatomical site and to maintain the drug concentration within a therapeutic band for a desired duration. Prolonged release of gentamicin from PLA thin film composites was studied and presented in Figure 3. Generally, the release of drug from biodegradable systems is rather complex phenomenon which can be governed by diffusion, dissolution, erosion and most often by a combination of these mechanisms. That is the reason why an adequate empirical or semi-empirical model that incorporates most of the release governing factors is required for the assessment of the release kinetics.

##### 2.1.4.1. Release Kinetics—Model Dependent Method

The release kinetic study was assessed by model dependent method. Based on number of kinetic models available in literature, which described the overall release of drug from the dosage forms, were carefully selected and used to fit the release data [11,12,13,14]. Finally, data were fitted to seven different models: Zero order, 1st Order, Higuchi, Hixson-Crowell, Korsmeyer-Peppas and Reciprocal powered time.

The correlation coefficients (*r*^2^) indicate that drug release kinetic of PLAGM and PLAHApGM fitted with the power law model described by Korsmeyer-Peppas (Table 1). If this semi-empirical equation doesn’t allow for the determination of all of the mechanisms involved in the release, it is still possible to determine the mechanisms of transport by considering two borderline cases, which correspond to distinct physical realities (when *n =* 0.5 and *n =* 1). The n coefficient obtained for PLAGM (*n* < 0.5) indicates that the release mechanism was mainly controlled by diffusion while the value obtained for PLAHApGM (*n* > 1) is characteristic of number of mixed transport mechanisms including diffusion, possibly super case II kinetics as well as a release due to damage to the composite surface through dissolution.

**Table 1:** Modelled dissolution characteristics of the mean dissolution profile.

Model	Mathematical Expression		PLAGM	PLAHApGM
Zero order	$F=Q_{0}+k_{0}t$	$r^{2}$	0.440	0.937
		*k*_0_	0.125	0.070
First order	Ln(1−F)=−kt	$r^{2}$	0.866	0.874
		*k*	0.226	0.098
Higuchi	$F=at^{1/2}+b$	$r^{2}$	0.959	0.803
		*a*	0.273	0.214
Hixson- Crowell	$1-\;{\left(1-F\right)}^{1/3}=kt$	$r^{2}$	0.758	0.898
		*k*	0.060	0.029
Korsmeyer-Peppas	$F=kt^{n}$	$r^{2}$	0.992	0.962
		*n*	0.282	1.315
Baker Lonsdale	$\frac{3}{2}\left[1-{\left(1-F\right)}^{2/3}\right]\times F=kt$	$r^{2}$	0.945	0.727
		*k*	0.107	0.038
Reciprocal powered time	$\left(\frac{1}{F}-1\right)=\frac{m}{t^{b}}$	$r^{2}$	0.971	0.835
		*b*	0.744	1.037
		*m*	1.524	17.190

*F* = fraction of drug released up to time t, *r*^2^ = Square correlation coefficient, *Q*_o_, *k*_0_, *k*, *a*, *b*, *n*, *m* and b are parameters of the models.

##### 2.1.4.2. Release Kinetics—Comparison of Drug Release Profiles

PLAGM and PLAHApGM dissolution profiles were compared using statistical difference factors, difference factor (f1), and similarity factor (f2) (Table 2). The test indicates that there is a significant statistical difference between the two kinetic profiles. This difference is confirmed by the release half-life (t_50%_) obtained for each material loaded with gentamicin. Thus, the release half-life of PLAGM is obtained around 14 days while 124 days are necessary for PLAHApGM.

**Table 2:** Modelled dissolution characteristics and difference and similarity factors of PLA film and PLAHAp composites loaded with gentamicin.

	PLAGM	PLAHApGM
t_50%_ (weeks)	1.76	15.53
f1 difference factor	54.4	
f2 similarity factor	24.2	

#### 2.1.5. Antibacterial Efficacy Test

**Figure 4:**
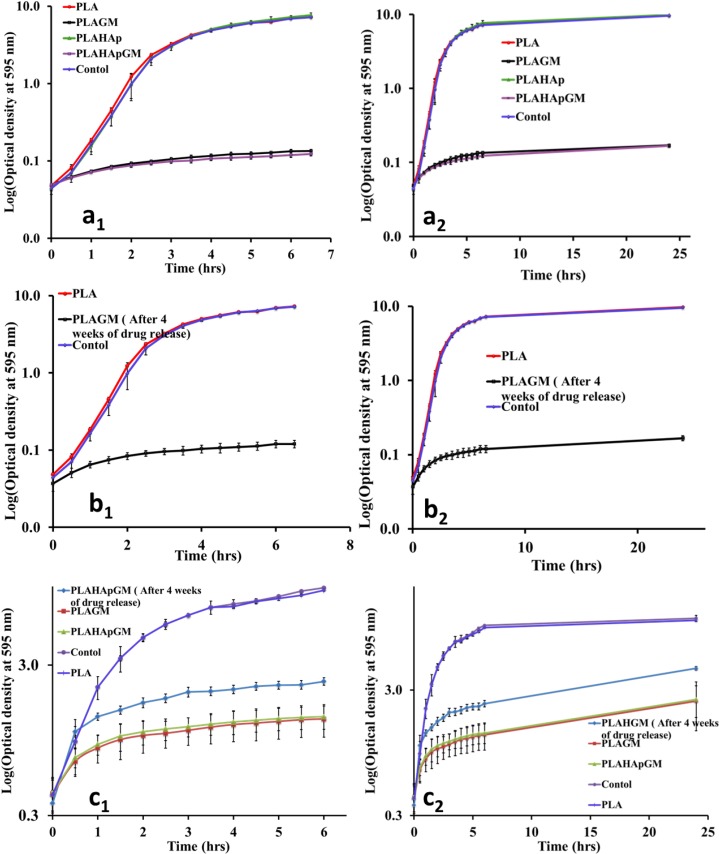
Antibacterial efficacy (**a_1_**) Controls compared with films loaded with gentamicin. All films were introduced immediately after inoculating media with bacteria; (**b_1_**) PLA films loaded with gentamicin after releasing gentamicin for four weeks in PBS (pH 7.4, 37 °C and 100 rpm). Plain PLA films and media were used as positive and negative control, all films were introduced immediately after bacteria inoculation; (**c_1_**) Films loaded with gentamicin and PLAHApGM film after releasing gentamicin for four weeks. Plain PLA films and media were used as positive and negative control; all films were introduced immediately after bacteria inoculation. **a_2_**, **b_2_**, and **c_2_** are post-bacterial growth level up to 24 h of respective experiment. Error bars are mean standard deviation (SD) of two biological and technical replicates of the experiments conducted in different days.

Drug loaded PLA composite films were introduced to a 50 mL subculture of *S. aureus* in the beginning and also in time-delay of 90 min where the bacteria growth is at the middle of log phase. In both cases, the released gentamicin concentration was enough to control the growth of bacterial as shown in Figure 4a–c. It was also revealed that after 24 h there was no bacterial recovery seen. Most of the drug released in this period of time is surface associated drug molecules. Furthermore, samples from release study, after four weeks of release were also collected and subjected to efficacy testing. This was important specifically when it is intended to be used as drug delivery system during prolonged periods.

#### 2.1.6. Morphology of Film Composites in Release Study

**Figure 5:**
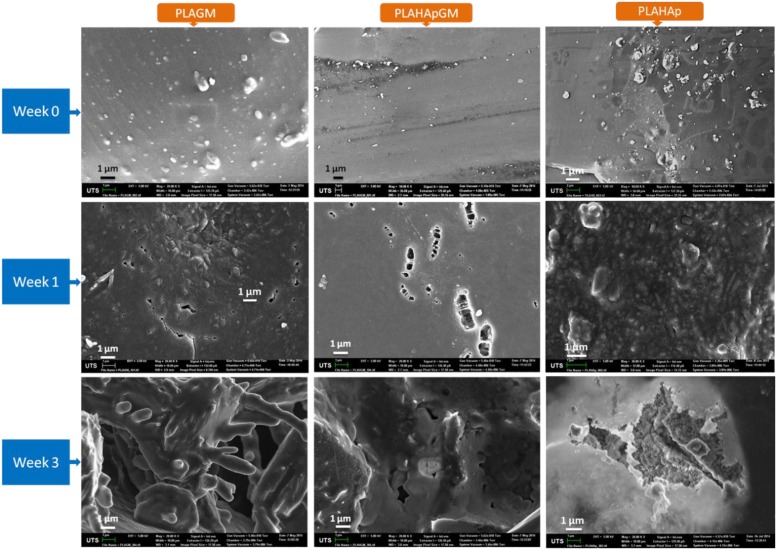
SEM pictures of PLAGM, PLAHApGM and PLAHAp composites revealing the degraded morphology after 1st and 3rd weeks in PBS solution.

The morphologies of the films were investigated by SEM. For extended drug controlled release systems, degradation of polymeric matrix and the additional particulate matter (microspheres) control the release of drug. Drug release rate and release time is highly influenced by degradation kinetics of the degradable polymer which means the slower the degradation the longer the release time. Figure 5 represents the morphology comparison of films loaded with gentamicin in release study after 1st and 3rd weeks to zero time.

The morphology of PLAHAp, gentamicin loaded PLA and PLAHAp thin film composites show the loaded particles existed in agglomerate forms within PLA matrix with an average size of 100 nm to 1.5 μm for PLAGM and PLAHAp and 200 nm to 1 μm for PLAHApGM (Figure 5-week 0). However the distribution of drug and HAp drug loaded particles within PLA matrix seems to be good. The morphology also suggests that PLAGM sample has more gentamicin protruding close to the polymer surfaces compared to PLAHApGM which is inconsistence with drug encapsulation efficiency data. It also explains the differences in hydrophilicity between gentamicin and hydroxyapatite, the factor that influences interaction between them and PLA matrix. PLA, being hydrophobic should interact more with relatively more hydrophobic materials.

#### 2.1.7. Statistical Analysis-Multivariate Approach (MANOVA)

In order to improve understanding of the drug dissolution we analysed release data using multivariate approach statistical method. The idea is to compare drug release from different PLA thin film composites where the release is measured at several points over time. The assumptions were sampling time were equally spaced, release were normally distributed and data were complete with no missing observations. Statistical software, SPSS 21.0 for Windows was employed. Multivariate tests were conducted on Pillai’s Trace, Wilks’ Lambda Hotelling’s Trace and Roy’s Largest Root. The results show that release differences between PLAGM and PLAHApGM were very significant with ** *p* ≤ 0.01 while the effect of time on their release was highly significant with *** *p* ≤ 0.001.

### 2.2. Discussion

The release of gentamicin from these devices seem to follow semi-empirical equation describe by Korsmeyer-Peppas model. Nevertheless, the ‘n’ coefficient obtained for PLAGM and PLAHApGM indicate that somehow a number of different mechanisms might control release. Thus, the release of gentamicin contained from PLA matrix seems to be mainly controlled by diffusion whereas for PLAHApGM is possibly mixture of diffusion, super case II mechanism and other mechanisms of transport which control drug release.

It should be added that the analysis shows that the diffusion mechanism is observed for the PLAGM model (regression coefficients of models which use diffusion are good). However it is certain that at the end of the initial release, degradation of the polymeric network will “favour” the dissolution of the remainder of the drug. So, this phenomenon (diffusion and degradation) should occur at the end of the surface drug release (when dissolution of drug have created secondary porosity inside the polymeric network and resultant “fragility” of it) and/or depends to the dissolution rate, surface area, of the PLA film used. For this reason, we have to consider only the early part of the dissolution kinetic (just below 60% drug released) and hence, in the case of PLAGM, the release can be assumed to proceed and mainly controlled by diffusional process.

However, for the PLAHApGM composite model, the transport mechanisms are more complex: firstly, initial PLA matrix dissolution proceeds which exposes the HAp particles, consequently the surface drug from these particles are dissolved. Coralline HAp as a structure contains meso and nanopores and during the initial drug loading period which utilises vacuum, gentamicin penetrates the pores of the “HAp-coral” and coats its surfaces and its porous network. The gentamicin contained in these porosities will be released gradually.

Following this initial PLA dissolution, HAp particle dissociation proceeds and further drug is released by diffusion. The process involves both diffusion and other transport mechanisms based on dissociation mode which accelerates the dissolution rate and hence influences the drug release rates. For this second mode we can assume that the release occurs in several stages:

1. Release of the drug contained on the exposed composite surface.
2. Diffusion of the drug through the polymeric network and slow release of some part of drug contained into the surfaces of the HAp particles which is less accessible. The polymer plays a role of barrier that allows a slower release.
3. Degradation/erosion and/or fragmentation of the polymeric composite which promotes accessibility to the “HAp-particle” porosity and facilitate release of gentamicin located inside porous network.
4. Degradation of the HAp particles, which facilitates dissolution of Ca^2+^ and PO_4_^3−^ ions and exposure of further GM and dissolution by diffusion to the environment.

Despite the fact that the mechanisms that govern the drug dissolution are not the same, diffusion might be the dominating mechanism in all of the mentioned processes. The release kinetic profiles of these biocomposites are characteristics of those of prolonged release drug delivery system. Dissolution profiles show slower gentamicin release from PLAHApGM highlighted by the t_50%_, 9 times higher than those of PLAGM. Moreover, the decrease of the rate of release is accompanied by the suppression of the early burst effect recorded for the PLAGM mixtures.

PLAHApGM shows more controlled release and it displays the potential from extended release applications. The main reason for this pattern is the fact that drug entrapped within hydroxyapatite particles takes longer time to dissolve and diffuse into the solution compared to drug in polymeric matrix. The release data in Figure 4 show that about 40% of drug was released after two weeks from PLAGM device while 20% of drug was released after 3 weeks in PLAHApGM. After the drug close to the polymer composite surfaces is released, further release rate will depend on the polymer degradation rate. Figure 5 shows progressive degradation of both PLAGM and PLAHApGM films after 1 and 3 weeks. Degradation of these films correlates with the drug release data with PLAGM showing faster degradation. Gentamicin being hydrophilic, PLAGM is suggested to be more hydrophilic and therefore more water interaction and resultant diffusion into the device and hence faster degradation and release rates. It is believed that after a period of time, degradation of PLA will be auto catalysed by the degraded acidic end groups. On the other hand, though PLAHAp film degraded progressively it seemed to be the slowest due to its relatively high hydrophobicity in the absence of drug.

The presence of hydroxyapatite as bioactive particles within the matrix has an extra advantage as calcium and phosphate ion source which widens its applications in tissue engineering particularly in maxillofacial and orthopaedic applications.

Clinical studies for systemic treatment of gentamicin suggest maximal antimicrobial activity is archived in once-daily gentamicin dosing of 10 times MIC. However, the studies also suggest that once-daily gentamicin treatment has similar toxicity to conventional multiple-daily administration. On the other hand, controlled release administration would significantly reduce toxicity and side effects of gentamicin in patients since only small amount of drug would be needed for local administration.

In this current study a bacterial culture, *S. aureus* (SH1000), was used for antibacterial efficacy of gentamicin loaded PLA-HAp marine based composites. Since gentamicin lacks chromophores, it doesn’t absorb light in either UV or visible spectrum; therefore it didn’t interfere with optical density measurements, the method used to monitor bacterial growth. The release concentration from PLAGM and PLAHApGM for several weeks, indicated to have enough antimicrobial activity as shown in Figure 4b, c. The results, also indicated that PLA thin film and PLAHAp film composite without gentamicin showed no significant differences compared to the control, media without films. The prolonged ability to release drug from the films was tested by subjecting films that have released gentamicin in PBS for four weeks into antibacterial efficacy testing. The results suggested that, even after releasing drugs for four weeks, films were able to still release enough gentamicin to prevent microbial activities, (Figure 4b,c). All films loaded with gentamicin exhibited significant ability to prevent bacterial growth even at high concentration of bacterial, first grown for 90 min (as shown in Figure 4c). The overall results indicate that no bacterial recovery observed in the culture with loaded films after 24 h.

## 3. Experimental Section

### 3.1. Materials

Corals were obtained from the Great Barrier Reef, QLD Australia. Gentamicin, diammonium hydrogen phosphate (NH_4_)_2_HPO_4_, 98%), and sodium hypochlorite (NaClO) were obtained from Sigma Aldrich, Castle Hill, Australia). Bacteria *Staphylococcus aureus* (SH1000) derived from a sepsis isolate (NCTC8325) was used in all experiments.

### 3.2. Methods

#### 3.2.1. Hydrothermal Conversion of Coral

The coral samples were crushed in a mill and cleaned with 2% (v/v) NaClO, further ground within an aluminium oxide ball mill (46 rpm, 2 h), and then dried at 100 °C for 2 h before use. Hydrothermal conversion was carried out following Ben-Nissan procedures [4]. Briefly, coral powder was converted in a Parr reactor at 250 °C with the stoichiometric amount of diammonium hydrogen phosphate (NH_4_)_2_HPO_4_ to obtain hydroxyapatite (HAp). Hydrothermally converted coral powders were loaded with gentamicin using rotary evaporation technique then dried in vacuumed desiccators and then used for thin film composites. The required amount of gentamicin was dissolved in polished 18 MΩ (MilliQ, Millipore, Victoria, Australia) water and then mixed with HAp powder in a sonicator for five minutes. Then the mixture was allowed to dry in rotary evaporator at 60 °C under vacuum.

#### 3.2.2. Preparation of Film Composites

PLA thin films and composites were prepared using a solvent casting technique described previously [10,15]. Briefly, PLA films were prepared by dissolving 0.50 g of polymer in 30 mL of chloroform (1.12% wt/v polymer concentration) under magnetic stirrer at room temperature until it was completely dissolved. Then the solution was transferred into petri dish and chloroform was allowed to evaporate under low vacuum (in a desiccator) for 48 h. The films were stored in desiccators for further analyses. The same procedure with 15 min ultrasonication was employed to prepare PLA with gentamicin (PLAGM) and PLA with converted coral and gentamycin (PLAHApGM) composites samples. Based on previous research [7,16] a range of gentamicin concentrations 5%–30% have been used. In any case minimum amount of drug should be considered for local release systems. A gentamicin concentration of 10% (w/w) was used in all experimental samples.

#### 3.2.3. Minimal Inhibitory Concentration and Antimicrobial Efficacy

Experimental samples used were designated as the following: Polylactic acid film as PLA: PLA film loaded with Gentamicin as PLAGM: PLA-hydroxyapatite composite film as PLAHAp: composite films of hydroxyapatite loaded with Gentamicin with PLA as PLAHApGM.

According to CLSI, the Minimal Inhibitory Concentration (MIC) is the lowest concentration of an antimicrobial agent that prevents visible growth of a microorganism in an agar or broth dilution susceptibility test [15,16] Gentamicin MICs were determined using 96-well microtitre trays and broth microdilution techniques. Inoculum concentration of 5 × 10^6^ CFU/mL was used in each well. MICs were determined in accordance with CLSI [17] guidelines. *S. aureus* ATCC 25923 strain was also used as the control *S. aureus* strain.

An antimicrobial efficacy test was conducted in Tryptone Soya Broth (TSB). Inoculum density equivalent to 0.5 McFarland standards (1 × 10^8^ CFU/mL) from overnight culture, with a 50 mL TSB sub-culture was used. PLA film composites loaded with gentamicin were either introduced in the beginning or after 90 min of bacterial growth in a dry incubator at 37 ± 0.1 °C, with shaking at 220 rpm. The optical density of the culture was monitored by spectrophotometer at 595 nm from the experiment at time 0, which was immediately after introduction of gentamicin loaded PLA films, and every half an hour for the first 6 to 6.5 h. When OD reaches about 1, samples were diluted to 1:10 before measurement. An additional time-point was obtained after 24 h to check for recovery. Replicates of all experiments were done in different days.

#### 3.2.4. Drug Release and Release Kinetics

Drug release study of PLA thin film composites was conducted under SINK conditions in phosphate buffered saline (PBS) (0.1 M, Na_3_N 0.1%, pH 7.4) at 37 ± 0.1 °C) in a temperature controlled water bath shaker running at constant speed of 100 rpm. Each sampling time had its own independent samples under the same conditions and experiments were respectively individually terminated after sampling. All drug concentrations were determined by Cary 100 UV-Vis spectrophotometer (Agilent Technologies, Victoria, Australia, Cary Series UV-Vis Spectrophotometer) at the maximum absorbance of gentamicin-*o*-phthaldialdehyde complex, λ_max_ = 332 nm, using procedures described in [7,18]. Ophthaldialdehyde reagent was prepared by dissolving 2.5 g ophthaldialdehyde in 62.5 mL methanol and adding with 3 mL 2-hydroxyethylmercaptan to 560 mL 0.04 M sodium borate in distilled water. 2 mL gentamicin solution, 2 mL *o*-phthaldialdehyde reagent were reacted for 45 min at room temperature. The absorbance, which corresponds to the gentamicin concentration, was then measured at 332 nm. A calibration curve was prepared for each set of measurements. Each prescribed sampling time had its own independent sample, therefore after sampling the respective experiment was terminated.

To study the mechanisms involved in the release of gentamicin associated with these composites, the results were fitted to various mathematical models, characterizing diffusion, dissolution or/and erosion prevalence as well as mix of dissolution-diffusion rate processes. Zero order, 1st Order, Higuchi, Hixson-Crowell, Korsmeyer-Peppas and Reciprocal powered time have been tested. The mathematical modelling was carried out using GraphPad Prism software (GraphPad software Inc., La Jolla, CA, USA). The comparison of the release profiles between PLAGM and PLAHApGM was evaluated using t_50%_ determined from the reciprocal powered time equation (*i.e.*, the time necessary to release 50% of the drug substance) and the difference (f1) and similarity (f2) factors [13]. Two dissolution profiles are considered similar when f1 is not greater than 15 and f2 varies from 50 to 100.

#### 3.2.5. Morphology and Mechanical Properties

The morphological analyses of PLA films and composites were performed in a Scanning Electron Microscope (SEM) (ZEISS Supra55VP, Zeiss, Germany). Samples were fixed by conductive adhesive tape on aluminium stubs and covered with carbon using a sputter coater. Images were taken at various magnifications at acceleration voltages of 20 kV.

Mechanical characterization was performed according to the method described in [10]. Drug encapsulation efficiency (DEE) was determined by soaking 2 mg of samples in 2 mL of distilled water at 37 °C for 5 min and then measured the drug concentration of the solution using UV-Vis. This process dissolves drugs that are on the outer surface of the films. The efficiency was determined by the formula Equation (1).

(1) $$DEE=\frac{W_{df}-W_{ds}}{W_{df}}$$

where, $W_{df}$ = Weight of drug in the film, $W_{ds}$ = Weight of drug dissolved in solution.

### 3.3. Statistical Analysis

All data were expressed as mean and standard deviation. The release data were compared using Multivariate test on Pillai’s Trace, Wilks’ Lambda Hotelling’s Trace and Roy’s Largest Root and differences were considered significant when * *p <* 0.05, very significant ** *p <* 0.01 and highly significant *** *p <* 0.001, respectively. A p-value higher than 0.05 (*p* > 0.05) was taken as indicating no significant difference [19].

## 4. Conclusions

The study of PLA–calcium phosphate thin film composites reveals possible potential applications of these devices in the biomedical field as drug delivery systems. The flexibility of these devices allows them to conform into any desired clinical shape and size. Incorporation of hydroxyapatite in this drug from hydrothermally converted “marine” materials, has added advantages of controlled release, improved encapsulation efficiency, increases drug stability and maintenance of bioactivity and continuous supply of calcium and phosphate ions which can assist in bone regeneration and repair. The release profiles, exhibited a steady state release rate, with significant antimicrobial activity even at high concentration of bacteria. The systems also showed the potential for prolonged release of antibiotic.

The developed systems have the potential to be applied to prevent microbial adhesion to medical implant surfaces due to its propensity to uptake and release antibiotics, as a consequence of its biodegradability potential. In addition, they can generally be used in surgery to prevent infections mainly caused by *S. aureus*. Characterization of *in vivo* release and bacterial adhesion on the film composites are being currently conducted.
